# Passive Downhole Pressure Sensor Based on Surface Acoustic Wave Technology

**DOI:** 10.3390/s17071635

**Published:** 2017-07-15

**Authors:** Sully M. M. Quintero, Sávio W. O. Figueiredo, Victor L. Takahashi, Roberth A. W. Llerena, Arthur M. B. Braga

**Affiliations:** Mechanical Engineering Department, Pontifical Catholic University of Rio de Janeiro, Rua Marquês de São Vicente 225, 22453-900 Rio de Janeiro, Brazil; saviowes@hotmail.com (S.W.O.F.); takahashi@puc-rio.br (V.L.T.); roberan@puc-rio.br (R.A.W.L.); abraga@puc-rio.br (A.M.B.B.)

**Keywords:** pressure sensor, downhole application, SAW device, wireless sensor

## Abstract

A passive surface acoustic wave (SAW) pressure sensor was developed for real-time pressure monitoring in downhole application. The passive pressure sensor consists of a SAW resonator, which is attached to a circular metal diaphragm used as a pressure transducer. While the membrane deflects as a function of pressure applied, the frequency response changes due to the variation of the SAW propagation parameters. The sensitivity and linearity of the SAW pressure sensor were measured to be 8.3 kHz/bar and 0.999, respectively. The experimental results were validated with a hybrid analytical–numerical analysis. The good results combined with the robust design and packaging for harsh environment demonstrated it to be a promising sensor for industrial applications.

## 1. Introduction

Throughout the last few decades, the use of permanent well monitoring systems has been experiencing a remarkable growth in the oil and gas industry [1]. Information such as downhole pressure and temperature are valuable in order to optimize production and also help in identifying or avoiding problems that may lead to an undesired shut-down of an oil well. For a long time now, technologies based on quartz crystals and fiber optics have dominated the market for downhole pressure gauges. Both require the use of downhole cables (electrical or optical), which increases the cost of the monitoring solution. With the downturn in the oil and gas industry, with oil prices expected to remain well below where they were in the first half of the decade, any solution that provides cost reductions is welcomed by oil producers. As a consequence, the use of downhole wireless telemetry has been gaining increased attention in the last few years [2,3,4,5,6]. However, in order to reach its full potential, wireless solutions will require low power consumption, opening a window of opportunity for surface acoustic wave (SAW) devices in monitoring systems for oil wells. This paper presents a passive SAW-based pressure sensor developed for these applications.

The first pressure sensor based on SAW was proposed in 1975 by Reeder et al. [7]. It consisted of SAW delay line transducers fabricated on the surface of a pressure-dependent membrane made of ST-X quartz. It was integrated into an oscillator, and its phase and time delay changed linearly with the applied pressure. Some years later, in 1997, Buff et al. [8] proposed a similar pressure sensor design, but using a SAW resonator instead of a delay line. This design used 36° Y cut quartz and could store electromagnetic energy captured with an antenna, thus being a passive remote pressure sensor. The versatility of SAW devices was also used to measure soil pressure, as addressed in 2014 by Wang et al. [9], who proposed a passive wireless SAW sensor consisting of a hollow cylinder with a sealing membrane in which the SAW transducers were fabricated, producing a delay line. A more current and successful employment of SAW pressure sensors is in the automotive industry in conjunction with tire pressure monitoring systems (TPMS). The SAW sensors are integrated to the vehicle’s wheels and communicate wirelessly with a central transponder. These sensors require no battery, and are what is called the second generation of TPMS [10]. The first generation TPMS was also based on Micro-Electro-Mechanical Systems (MEMS) pressure sensors and required battery to supply energy to an active transmitter [11].

Alternative materials other than quartz have been investigated for pressure sensor fabrication. In 2013, Rodriguez-Madrid and Iriart [12] developed a high-frequency (GHz) SAW pressure sensor for gases made from aluminum nitrate (AlN) as the piezoelectric material deposited on nanocrystalline diamond (NCD) membrane structure. The materials allowed the sensor to be used in high-temperature environments, and has achieved extreme pressure sensibility. Aiming at reducing the production cost of SAW pressure sensors, S. Grousset et al. [13] depicted a wafer-level approach to transfer single-layer Quartz crystal onto Silicon substrate. This allowed for batch production, better control of all sensors characteristics, and better integration with the electronics.

While the majority of SAW pressure sensors have been designed as membrane-like devices, in 2014, Della Lucia et al. [14] proposed a different way to use SAW devices for this application. The essence of their design consisted of subjecting a SAW device to compression loading instead of bending loading such as the one a membrane is subjected to. The packaging comprised a capsule where the device was fixed and compressed with the aid of a mechanical plunger connected to a membrane. Under pressure, the membrane presses the plunger, which in turn compresses the SAW device. This pressure sensor was mentioned to be used in the oil offshore and gas industry, and was able to measure pressures ranging from 0 to 1000 bar—far above the upper pressure limits of the aforementioned designs.

As can be noticed in Table 1, the maximum operating pressure capability reported by most researchers is generally low. Since the mechanical strength of the piezoelectric materials used is relatively low, the sensors cannot be used to measure high levels of pressure. This is a limiting factor when one desires to use these sensors in high-pressure environments like inside a wellbore, where pressure conditions may reach up to 2700 bar in extreme cases [15]. 

In order to measure pressure values in the vicinity of 100 bar with relatively enhanced sensitivity, we propose an alternative way to deploy SAW devices for pressure measurement. By bonding a SAW resonator on a metallic membrane previously designed to stand to pressures of up to 200 bar, the mechanical strength limitation of quartz membranes is overcome. The strain levels in the membrane do not reach harmful values, thus the SAW device is kept safe. The design also includes passive and wireless operation of the sensor.

## 2. Design and Numerical Simulation

### 2.1. Design

The working principle of this sensor is the detection of the strain produced by the applied pressure on a circular diaphragm instrumented with a SAW resonator. The pressure sensor is mounted directly on a mandrel specifically developed to accommodate up to two transducers. The diaphragm was designed so as to remain uncoupled from the deformations of the mandrel. 

**Figure 1:**
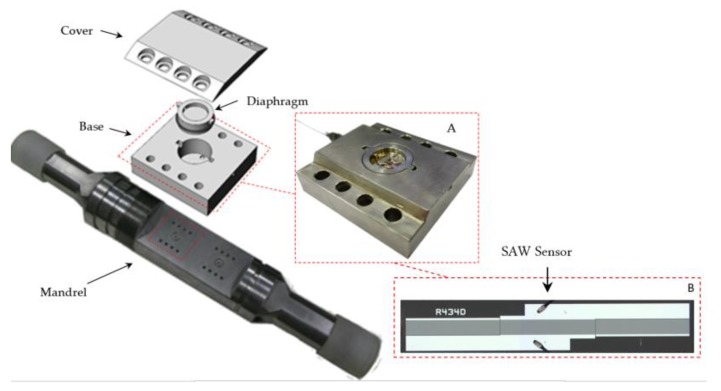
Pressure sensor assembly. In detail **A**: diaphragm mounted in the base. In detail **B**: top view of the SAW sensor.

As depicted in Figure 1, the pressure sensor consists of a base, a diaphragm, a cover, and a SAW resonator. All metallic parts, designed and machined in our laboratory, were made of stainless steel AISI 316, except the diaphragm, which was made of Inconel™ 718. The material of the diaphragm was thermally treated to achieve its maximum strength (precipitation hardening at 718 °C). Figure 1 also shows the mandrel designed to accommodate the pressure sensor. The dashed red line points out the place where they are to be placed. The main parts of the sensor are schematically shown in the exploded view. The detail “A” shows the picture of the base with the diaphragm already instrumented.

The base has grooves for O-rings and backup rings (made from a fluoroelastomer marketed under the Viton^®^ brand). These O-rings have two roles: the first is to isolate the lower chamber of the diaphragm, on which the pressure to be measured acts, from the upper chamber instrumented with the SAW resonator; the second is to isolate the diaphragm from the deformations coming from the base and cover of the transducer, and to avoid position offset. Regarding the cover, it keeps the diaphragm from detaching and protects the SAW from any possible contamination. The SAW device shown in the detail “B”, attached to the upper surface of the diaphragm, is a one-port configuration resonator operating at 433.92 MHz. It consists of one interdigital transducer (IDT) and two banks of reflectors on a ST-cut quartz piezoelectric substrate with the acoustic aperture of 0.2 mm and pitch of 1.5 µm. Basically, the IDT is a series of interleaved metallic electrodes responsible for generating the surface waves.

### 2.2. Numerical Simulations

Although the strain distribution in a rigidly clamped diaphragm under uniform pressure is well understood, we have chosen to perform numerical simulations in order to better understand the distribution and effective deformation transferred from the diaphragm to the SAW resonator. We employed ANSYS Workbench 16.0 finite element analysis (FEA) software to analyze the strains transferred to the SAW device when the diaphragm is subjected to pressures of up to 100 bar. The following parameters were used as input in the simulations:

- Diaphragm: modeled as AISI 316 steel, with thickness and diameter of 0.6 and 15 mm, respectively.
- Adhesive layer: considered to be a 30 µm-thick isotropic material with Young’s modulus of 3.5 GPa.
- SAW resonator: 0.7 mm wide, 3.6 mm long, and 0.35 mm thick, and modeled as ST-cut quartz material, with the wave propagation direction parallel to radial direction.

Every domain was meshed with tetrahedral elements—specifically the element SOLID187, which is a 3-D 10-node tetrahedral. The total number of nodes in the FE model was 411128. 

**Figure 2:**
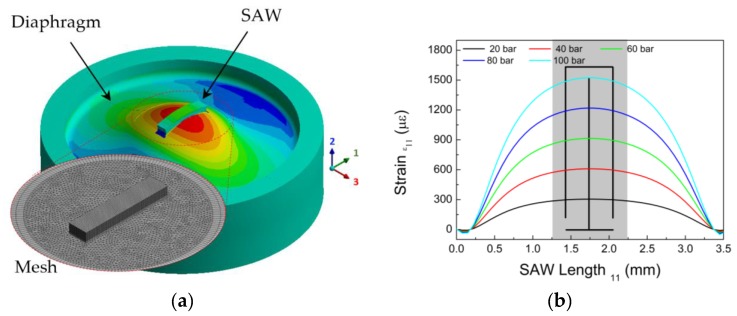
(**a**) Radial strain distribution (in the inset: mesh density close to the SAW device). (**b**) Radial strain distribution on the top surface of the SAW device at increasing pressures.

Figure 2a shows the radial strain field on the diaphragm-SAW system, and in the inset, the detail of the mesh surrounding the SAW. Figure 2b shows the radial strain distribution along the longitudinal axis of the SAW device for different pressure values, at the upper surface. The grey shaded area corresponds to the IDT region, from which the surface waves are launched. The maximum strain variation between the center point and the IDT edge is approximately of 6% of the full scale.

## 3. Experimental Setup 

In order to characterize the sensor’s response to pressure, the sensor was screw-fastened to a pressure vessel with identical docking to that of the mandrel designed for field test. For this purpose, a calibrated dead-weight tester purchased from DH-Budenberg (580) (Salford, Manchester, England) and a pressure gauge purchased from Fluke (700P29) (Everett, WA, USA) were used, respectively, as the standard and pressure reference. The resonance frequency was monitored using a vector network analyzer (VNA) purchased from Keysight (E5061B) (Bayan Lepas, Penang, Malaysia).

**Figure 3:**
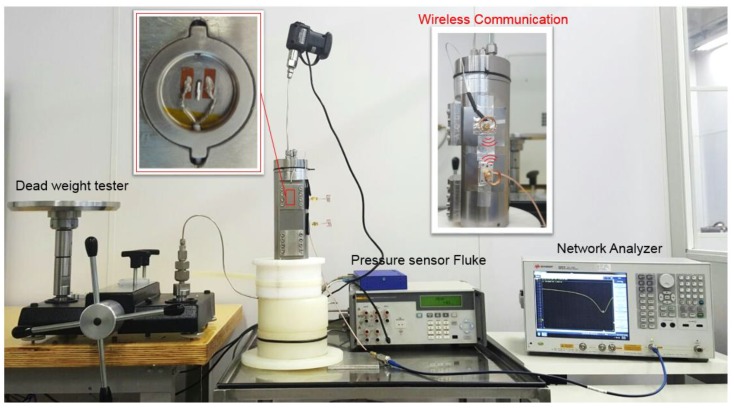
Setup for the pressure tests: in the left inset, the diaphragm instrumented with the SAW; at the center of the figure, the side view of the mandrel with antennas; and in the top-right inset, a plot of one of the load cycles.

The pressure test setup is shown in Figure 3. The dead-weight tester’s port is connected to the inlet port of the mandrel. The mandrel's outlet port is connected to the pressure gauge. In one of the insets in the picture, it is possible to see the antennas used for the wireless performance tests. The wireless arrangement consists of a pair of custom dipole antennas with an offset of 5 cm, using the mandrel as the ground plane. The output power and the number of points of the VNA was set to 10 dBm and 1601, respectively.

In addition, the left inset picture shows the top-surface of the diaphragm instrumented with the SAW resonator.

All experiments were performed at room temperature (25 °C). The pressure load was increased step by step, in about seven steps, up to a maximum of 96 bar. Each step was kept for 3 min and simultaneously monitored by the pressure gauge and the VNA. To test the sensor reproducibility, three load cycling tests were performed.

## 4. Results and Discussion

**Figure 4:**
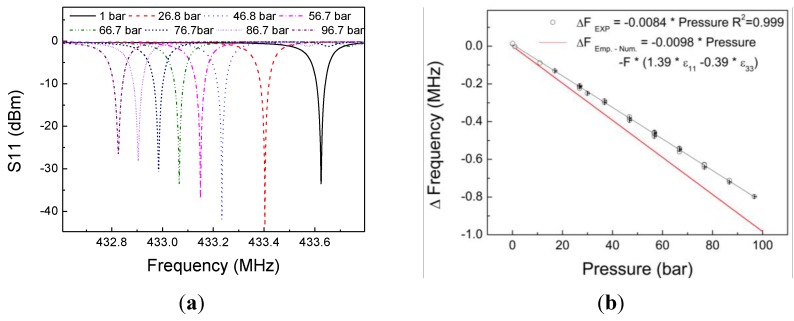
(**a**) S11 curve for all applied pressures. (**b**) Comparison of the variation of the SAW resonance frequency as a function of pressure calibration curve at ambient temperature (25 °C).

Figure 4a shows the frequency response of the sensor measured at different pressure levels (0 to 97 bar) and at the temperature of 25 °C. We clearly notice the shift in the resonance frequency as the applied pressure increases. The calibration curve for the sensor is reproduced in Figure 4b, which presents the frequency changes corresponding to scattering parameter S11. There is a linear dependence of the frequency on the pressure, with calculated sensitivity of 8.4 kHz/bar. This behavior is expected, since the diaphragm’s strain response to pressure is linear, and strain transfer occurs from the diaphragm to the SAW resonator. 

These results were compared to an analytical formula presented by Sinha et al. [16] for the behavior of the frequency of a SAW resonator undergoing biaxial strain. Their methodology considered the case of a circular piezoelectric disk subjected to normal force, where a SAW resonator was built directly on its top. In our case, the sensing element (SAW resonator) is attached to a circular metal diaphragm, which transfers flexural strain to the SAW resonator through the adhesive. The transferred strain induces changes in the pitch of the interdigital transducer, elastic constants, and density of the propagating medium. The expression for predicting the total fractional change in frequency may be given by the expression

(1) $$\frac{\Delta f}{f}=-1.39\;\varepsilon_{11}+0.39\;\varepsilon_{33}$$

where *f* is the reference frequency, $\varepsilon_{11}$ is the strain in the wave propagation direction, and $\varepsilon_{33}$ is the strain perpendicular to the direction of propagation. The corresponding $\varepsilon_{11}$ and $\varepsilon_{33}$ were obtained from the numerical simulations described in Section 2.2. The red curve in Figure 4b, based upon the above equation, is almost 20% more sensitive to strain. The disagreement between our experimental results and those obtained by Sinha et al. [16] may be related to the lower strain transfer performance of the sensor configuration. Strain in the diaphragm is partially absorbed by the adhesive when it is transferred to the SAW resonator, and thus only a part of the total membrane’s strain is sensed.

In order to verify and understand these discrepancies, tensile specimens were instrumented with the same type of SAW and adhesive used in the development of the presented pressure sensor. In this case, the tensile results were compared to other analytical formula proposed by Sinha and Locke [17]. Similar discrepancies were observed, confirming that the disagreements are effectively associated to the strain transfer performance of the sensor configuration

**Figure 5:**
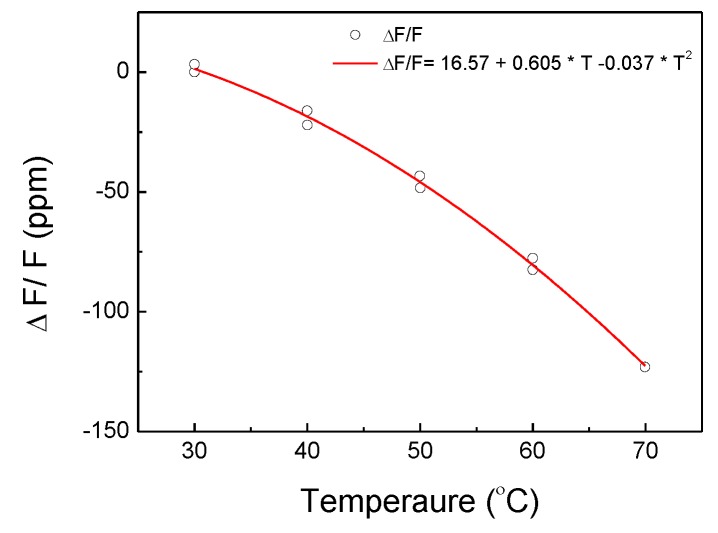
Variation of the SAW resonance frequency as a function of temperature (25 °C).

Finally, the sensor was characterized for temperatures ranging from 30 to 70 °C. Figure 5 shows a nonlinear response of the relative frequency drift as a function of the temperature. The square fitting indicates a second-order temperature coefficient value of −37 ppb/°C It is slightly higher than the second-order temperature coefficient for the ST cut quartz (i.e., about −34 ppb/°C). This slight difference may be a result of the interaction between materials with mismatched thermal expansion coefficients such as quartz, stainless steel, and the adhesive. 

## 5. Conclusion

Other authors have already demonstrated the applicability of pressure sensors based on SAW resonators; however, the low mechanical strength of quartz limits the sensors’ maximum operating pressure and restricts their applicability. In this paper, we proposed and demonstrated a wireless pressure sensor based on a SAW resonator with outstanding results with respect to the balance between operating range and sensitivity. The linear pressure sensitivity was estimated to be 8.3 kHz/bar, and the measured second-order temperature coefficient value was −37 ppb/°C.

Although the sensor was tested only up to 70 °C, the current design can be improved to allow for higher temperatures. One could use a SAW resonator fabricated on Langasite substrate, which has been proven to operate at a temperature of 500 °C [18], and bond it to the diaphragm using a ceramic cement adhesive. This adhesive type withstands high temperatures, and is successfully used to install resistive strain gages. This combination was demonstrated in the work of [19], which described the behavior of a high-temperature SAW strain sensor, and reported low hysteresis values. 

A numerical analysis in conjunction with an analytical formula was used to study the behavior of the frequency change under different strain distributions, and discrepancies were found. Initially, it was not clear whether the error stemmed from the hybrid analysis or the experimental measurements. However, the results of tensile tests confirmed that the disagreements were associated mainly with the performance of the strain transfer coefficient.

## Figures and Tables

**Table 1:** Summary of the main parameters related to pressure sensors based on surface acoustic wave (SAW) technology.

Author	Sensitivity	Range (bar)	Substrate	Technology	Wireless
Reeder et al. [7]	44–145 ppm/bar	0–3.5	ST-X Quartz	Delay Line	No
Buff et al. [8]	76 ppm/bar	0–10	35 Y Quartz	Resonator	Yes
Wang et al. [9]	330 kHz/bar	0–4	AIN on CVD Nanocrystaline	1-port Resonator	No
Rodriguez-Madrid and Iriart [12]	0.125 kHz/bar	0–1000	ST-X Quartz	2-port Resonator	No
Della Lucia et al. [14]	203 Hz/bar	0–1034	ST-X Quartz	2-port Resonator	No
Grousset et al. [13]	25.8 kHz/bar	0–4.8	AT-Cut Quartz (YXl)/37°	1-port Resonator	No
